# Blood pressure-lowering treatment strategies based on cardiovascular risk versus blood pressure: A meta-analysis of individual participant data

**DOI:** 10.1371/journal.pmed.1002538

**Published:** 2018-03-20

**Authors:** Kunal N. Karmali, Donald M. Lloyd-Jones, Joep van der Leeuw, David C. Goff, Salim Yusuf, Alberto Zanchetti, Paul Glasziou, Rodney Jackson, Mark Woodward, Anthony Rodgers, Bruce C. Neal, Eivind Berge, Koon Teo, Barry R. Davis, John Chalmers, Carl Pepine, Kazem Rahimi, Johan Sundström, on behalf of the Blood Pressure Lowering Treatment Trialists’ Collaboration

**Affiliations:** 1 Department of Medicine, Northwestern University, Chicago, Illinois, United States of America; 2 University Medical Center Utrecht, Department of Vascular Medicine, Utrecht, Netherlands; 3 National Heart, Lung, and Blood Institute, Division of Cardiovascular Sciences, Bethesda, Maryland, United States of America; 4 Population Health Research Institute, McMaster University, Hamilton, Canada; 5 The Istituto Auxologico Italiano, Milan, Italy; 6 Centre for Research in Evidence Based Practice, Bond University, Robina, Australia; 7 School of Population Health, Faculty of Medical and Health Science, University of Auckland, Auckland, New Zealand; 8 The George Institute for Global Health, University of Oxford, Oxford, United Kingdom; 9 The George Institute for Global Health, Sydney, Australia; 10 Department of Cardiology, Oslo University Hospital, Oslo, Norway; 11 School of Public Health, University of Texas, Dallas, Texas, United States of America; 12 Division of Cardiovascular Medicine, University of Florida, Gainesville, Florida, United States of America; 13 Department of Medical Sciences, Uppsala University, and Uppsala Clinical Research Center, Uppsala, Sweden; Scripps Translational Science Institute, UNITED STATES

## Abstract

**Background:**

Clinical practice guidelines have traditionally recommended blood pressure treatment based primarily on blood pressure thresholds. In contrast, using predicted cardiovascular risk has been advocated as a more effective strategy to guide treatment decisions for cardiovascular disease (CVD) prevention. We aimed to compare outcomes from a blood pressure-lowering treatment strategy based on predicted cardiovascular risk with one based on systolic blood pressure (SBP) level.

**Methods and findings:**

We used individual participant data from the Blood Pressure Lowering Treatment Trialists’ Collaboration (BPLTTC) from 1995 to 2013. Trials randomly assigned participants to either blood pressure-lowering drugs versus placebo or more intensive versus less intensive blood pressure-lowering regimens. We estimated 5-y risk of CVD events using a multivariable Weibull model previously developed in this dataset. We compared the two strategies at specific SBP thresholds and across the spectrum of risk and blood pressure levels studied in BPLTTC trials. The primary outcome was number of CVD events avoided per persons treated. We included data from 11 trials (47,872 participants). During a median of 4.0 y of follow-up, 3,566 participants (7.5%) experienced a major cardiovascular event. Areas under the curve comparing the two treatment strategies throughout the range of possible thresholds for CVD risk and SBP demonstrated that, on average, a greater number of CVD events would be avoided for a given number of persons treated with the CVD risk strategy compared with the SBP strategy (area under the curve 0.71 [95% confidence interval (CI) 0.70–0.72] for the CVD risk strategy versus 0.54 [95% CI 0.53–0.55] for the SBP strategy). Compared with treating everyone with SBP ≥ 150 mmHg, a CVD risk strategy would require treatment of 29% (95% CI 26%–31%) fewer persons to prevent the same number of events or would prevent 16% (95% CI 14%–18%) more events for the same number of persons treated. Compared with treating everyone with SBP ≥ 140 mmHg, a CVD risk strategy would require treatment of 3.8% (95% CI 12.5% fewer to 7.2% more) fewer persons to prevent the same number of events or would prevent 3.1% (95% CI 1.5%–5.0%) more events for the same number of persons treated, although the former estimate was not statistically significant. In subgroup analyses, the CVD risk strategy did not appear to be more beneficial than the SBP strategy in patients with diabetes mellitus or established CVD.

**Conclusions:**

A blood pressure-lowering treatment strategy based on predicted cardiovascular risk is more effective than one based on blood pressure levels alone across a range of thresholds. These results support using cardiovascular risk assessment to guide blood pressure treatment decision-making in moderate- to high-risk individuals, particularly for primary prevention.

## Abstract 2

In an individual participant data meta-analysis, Kazem Rahimi and colleagues compare blood pressure lowering treatment strategies based on cardiovascular risk versus blood pressure.

## Author summary

**Why was this study done?:**

- Hypertension treatment guidelines have traditionally relied primarily on blood pressure levels to guide use of blood pressure-lowering medications.
- Cardiovascular disease (CVD) prevention guidelines, like those for cholesterol management, instead advocate for multivariable CVD risk assessment to guide treatment decisions.
- Simulation studies have modeled the benefits of using CVD risk to guide blood pressure management, but there has not been a direct comparison using clinical trial data with actual outcome events.

**What did the researchers do and find?:**

- We included individual participant data from 11 trials (48,872 participants) of participants in the Blood Pressure Treatment Trialists' Collaboration (BPLTTC).
- We estimated the number of CVD events avoided for a given number of persons treated using CVD risk to determine blood pressure-lowering treatment compared with using systolic blood pressure (SBP) level.
- We compared the two treatment strategies at specific blood pressure thresholds and across the spectrum of CVD risk and SBP levels studied in the BPLTTC.
- We demonstrated that a treatment strategy based on predicted CVD risk could prevent more events and require treatment of fewer persons than one based on SBP level.
- Using predicted CVD risk was particularly beneficial in the primary prevention subgroup.

**What do these findings mean?:**

- These results support using cardiovascular risk assessment to guide blood pressure treatment decisions in moderate- to high-risk individuals.
- The results challenge current paradigms in hypertension management and highlight the merits of using predicted CVD risk to guide blood pressure-lowering treatment intensity.

## Introduction

Clinical practice guidelines for hypertension treatment have traditionally relied primarily on blood pressure levels to guide use of blood pressure-lowering medications [1–4]. However, single risk factor levels, like blood pressure, incompletely capture risk. Furthermore, blood pressure-lowering medications provide a fairly consistent relative risk reduction across a range of blood pressure levels, leading to large variations in absolute benefit from blood pressure treatment observed among individuals [5–7].

In contrast to hypertension guidelines, cholesterol treatment guidelines have moved away from single risk factor levels to guide treatment and instead advocate for multivariable absolute cardiovascular disease (CVD) risk assessment to guide treatment decision-making [8–10]. Updates to some cholesterol guidelines have moved even further by eliminating cholesterol goals altogether and identifying CVD risk thresholds to guide clinician–patient risk discussions about statin initiation in primary prevention [8,10].

Recently, an analysis from the Blood Pressure Lowering Treatment Trialists’ Collaboration (BPLTTC) demonstrated similar relative risk reductions from blood pressure-lowering medications across a range of predicted risk strata with correspondingly greater absolute risk reductions in those with higher predicted risk [11]. Those results provide support for the role of CVD risk assessment in guiding blood pressure-lowering treatment decisions. Although simulation studies have modeled the benefits of a CVD risk strategy for blood pressure-lowering treatment compared with traditional hypertension guidelines [12,13], to date, there has not been a direct comparison of the two strategies using clinical trial data with actual outcome events. Such evidence is needed to move CVD risk-based treatment strategies into clinical practice.

Individual participant data from trials in the BPLTTC provide an ideal opportunity to compare these two treatment strategies across a range of possible treatment thresholds in a group of individuals who were randomly assigned to blood pressure-lowering therapy in a clinical trial setting. In this study, we sought to compare outcomes using a treatment strategy based on predicted CVD risk (CVD risk strategy) with one based on systolic blood pressure (SBP) level (SBP strategy).

## Methods

This analysis followed a prespecified protocol that was presented to the BPLTTC Steering committee in April 2013.

### Trial eligibility and comparisons

This meta-analysis includes individual participant data from the BPLTTC from 1995 to 2013. Trials were eligible for this analysis if they met the original inclusion criteria for the Collaboration overviews and were part of the subset of trials that randomly allocated participants to blood pressure-lowering drug or placebo or to a more intensive versus less intensive blood pressure drug treatment regimen [14]. Eligible trials were also required to have a minimum of 1,000 patient years of planned follow-up in each randomized group and to have not presented their main results before the Collaboration protocol was finalized in July 1995 [14]. For this analysis, we analysed data from all eligible trials that provided sufficient information to enable absolute CVD risk estimation.

We included the following treatment comparisons: angiotensin-converting enzyme (ACE) inhibitors versus placebo; calcium channel blockers versus placebo; diuretics versus placebo; and more intensive versus less intensive blood pressure-lowering regimens (regardless of drug class). We combined these comparisons to maximize statistical power, since prior analyses have demonstrated that most of the treatment effects were dependent on the amount of blood pressure reduction achieved with few drug-specific effects [15].

### Outcomes

We analyzed outcomes prespecified in the original BPLTTC protocol [14]. Our primary outcome was total major CVD events, defined as a composite of stroke (nonfatal stroke or death from cerebrovascular disease), coronary heart disease (nonfatal myocardial infarction or death from coronary heart disease, including sudden death), heart failure (causing death or resulting in admission to hospital), or CVD death.

### Cardiovascular risk estimation

We used a multivariable Weibull model previously developed in this dataset from the placebo groups. This model uses age, sex, body mass index, systolic and diastolic blood pressure (DBP), prior blood pressure-lowering treatment, smoking status, diabetes mellitus status, and history of CVD to estimate 5-y CVD risk. Details of the derivation and validation of this model have been previously published [11].

### Cardiovascular events avoided by treatment

To estimate the number of CVD events avoided by the CVD risk and SBP strategies, we ranked all eligible participants by decreasing levels of baseline CVD risk and then by decreasing levels of baseline SBP. Next, we considered a potential treatment threshold for each percentile of CVD risk or SBP observed in the dataset. For each strategy, we assumed that all participants with a level above a given threshold would be treated and everyone below would be untreated. We then noted the number of persons above the threshold (“N treated”) and the Kaplan-Meier estimated 5-y risks in the control groups above the threshold (“untreated 5-y risk”) at each percentile for each treatment strategy. In the subgroup of participants above the threshold, we calculated the relative risk reduction from blood pressure-lowering therapy. For that purpose, we used a one-step meta-analysis approach, fitting Weibull models with shared frailty for each trial, thereby preserving the randomized structure of the trials [16]. To estimate the risk among the treated (“treated 5-y risk”), we applied the obtained hazard ratios to the Kaplan–Meier 5-y risk among control group participants above the threshold using the following formula: treated 5-y risk = 1 − exp(hazard ratio * ln(1 − untreated 5-y risk)). We thereafter applied this to the total number of persons above the threshold to calculate the number of events avoided with 5 y of treatment: avoided events = *n* treated * (untreated 5-y risk − treated 5-y risk)).

The number of events avoided was graphically presented against the number and proportion of treated persons. We then calculated the areas under the curves to estimate treatment effectiveness (i.e., number of events avoided per persons treated) for each strategy, where greater area reflects more events avoided per persons treated. Areas were calculated as integrals using the trapezoidal rule and were expressed as the ratio of the obtained area to the maximum possible area (maximum number of cardiovascular events avoided multiplied by the maximum number of persons treated); treatment given at random to half the sample without consideration of any treatment thresholds corresponding to an area of 0.5. We used bias-corrected 95% bootstrap estimates from 10,000 repetitions to generate confidence intervals (CIs). We also calculated numbers needed to treat for 5 y to avoid 1 cardiovascular event as 1/(number of events avoided/number of treated persons for 5 y) at each threshold for each treatment strategy.

### Secondary analyses

We evaluated the expected outcomes of each treatment strategy in subgroups based on presence or absence of previous blood pressure-lowering treatment, diabetes mellitus, and prevalent CVD. The risk equation was well calibrated in all subgroups (S1 Fig). In a 2-stage meta-analysis combining estimates in complementary pairs of subgroups, heterogeneity of results between subgroups was assessed using I^2^ with corresponding 95% CIs. To determine if any differences in cardiovascular events avoided were related to differences in magnitude of SBP reduction, we quantified mean observed SBP reduction with blood pressure-lowering treatment using a mixed-effects generalized linear model with the participant as the unit of analysis and a random intercept for trial. Using these estimates, we standardized hazard ratios for each threshold and for each treatment strategy to a 5 mmHg SBP reduction. Given the strong effect of age on 5-y predicted CVD risk, we performed a sensitivity analysis comparing the CVD risk strategy and SBP strategy with an age-based treatment strategy. For the age-based strategy, we ranked all eligible participants by decreasing levels of age in a manner similar to what was done for baseline SBP in the SBP strategy. Lastly, in a subsample without previous CVD, we performed a sensitivity analysis using the Framingham total CVD risk equation [17]. We recalibrated the published equation to the observed survival rates and risk factor levels (means and proportions) in the BPLTTC dataset, substituting body mass index when trials were missing lipid values.

We used Stata version 14 (Stata Corporation, College Station, TX, United States of America) for all analyses.

## Results

### Baseline characteristics

We included 11 trials consisting of 47,872 participants (35,671 participants without prevalent CVD) in our analysis (some trials were factorial or included more than two groups) (S5 Fig) [18–28]. Pooled baseline characteristics of participants are shown in Table 1. Mean systolic and DBP differences between the active/more intense treatment versus placebo/less intense treatment were 5.7/3.2 mmHg (95% CIs 5.5–6.0 mmHg and 3.0–3.3 mmHg, respectively). Baseline characteristics and achieved blood pressure reduction by trial are included in Table 2 and S1 Table.

**Table 1:** Baseline characteristics of participants from the BPLTTC (*n* = 47,872).

Characteristics	Active/more intensive blood pressure-lowering treatment	Placebo/less intensive blood pressure-lowering treatment	Total
Participants, *n*	21,021	26,851	47,872
Mean age, y (SD)	65.7 (9.7)	64.7 (9.3)	65.2 (9.5)
Women, *n* (percentage)	9,614 (46)	12,298 (46)	21,912 (46)
BMI, kg/m^2^ (SD)	27.6 (4.8)	27.8 (4.8)	27.7 (4.8)
Mean SBP, mmHg (SD)	158 (22)	161 (21)	160 (21)
Mean DBP, mmHg (SD)	91 (13)	94 (13)	93 (13)
Mean total cholesterol, mmol/L (SD)	5.5 (1.5)	5.7 (1.4)	5.6 (1.5)
Mean HDL cholesterol, mmol/L (SD)	1.4 (0.6)	1.4 (0.6)	1.4 (0.6)
Previous antihypertensive treatment, *n* (percentage)	11,977 (57)	15,073 (56)	27,050 (57)
Current smoking, *n* (percentage)	3,031 (14)	4,069 (15)	7,100 (15)
Diabetes mellitus, *n* (percentage)	8,048 (38)	8,225 (31)	16,273 (34)
Previous CVD, *n* (percentage)	6,051 (29)	6,150 (23)	12,201 (25)

Data obtained from participants in ABCD3_H, ABCD3_N, ADVANCE, BENEDICT1&2, HOT, HYVET, PART2, PREVENT, PROGRESS, SCAT, and SYST-EUR [18–28]. Numbers of patients (*n*) are unbalanced because participants in some trials were not randomly assigned in a 1:1 ratio. To convert from mmol/L to mg/dL for total cholesterol and HDL cholesterol, divide by 0.0259.

**Abbreviations:** ABCD, Appropriate Blood Pressure Control in Diabetics; ADVANCE, Action in Diabetes and Vascular Disease: Preterax and Diamicron MR Controlled Evaluation; BMI, body mass index; BENEDICT; Bergamo Nephrologic Diabetes Complications Trial; BPLTTC, Blood Pressure Lowering Treatment Trialists’ Collaboration; CVD, cardiovascular disease; DBP, diastolic blood pressure; HDL, high-density lipoprotein; HOT, Hypertension Optimal Treatment; HYVET, Hypertension in the Very Elderly Trial; PART2, Prevention of Atherosclerosis with Ramipril; PREVENT, Prospective Randomized Evaluation of the Vascular Effects of Norvasc Trial; PROGRESS, Perindopril Protection Against Recurrent Stroke Study; SCAT, Simvastatin/Enalapril Coronary Atherosclerosis Trial; SBP, systolic blood pressure; SYST-EUR, Systolic Hypertension in Europe.

**Table 2:** Characteristics of included trials.

Characteristic	ABCD		ADVANCE	BENEDICT		HOT	HYVET	PART2	PREVENT	PROGRESS	SCAT	SYST-EUR
	Hyper-tensive sample	Normo-tensive sample		ACEI	CCB							
Treatment regimen	*More versus less intense*	*More versus less intense*	*Perindopril* *+indapamide versus placebo*	*Trandolapril versus placebo*	*Verapamil versus placebo*	*More versus less intense*	*Indapamide (+perindopril) versus placebo*	*Ramipril versus placebo*	*Amlodipine versus placebo*	*Perindopril (+indapamide) versus placebo*	*Enalapril versus placebo*	*Nifedipine versus placebo*
Participants, *n*	470	479	11,133	453	454	18,776	3,719	617	822	6,074	240	4,635
Mean age, y (SD)	57.8 (8.3)	59.3 (8.3)	65.8 (6.4)	62.0 (8.1)	62.5 (8.2)	61.5 (7.5)	83.5 (3.2)	60.5 (8.1)	57.1 (9.6)	63.9 (9.6)	60.8 (8.7)	69.7 (6.7)
Women, *n* (%)	153 (33)	218 (46)	4,732 (43)	221 (49)	210 (46)	8,875 (47)	2,255 (61)	114 (18)	163 (20)	1,845 (30)	28 (12)	3,098 (46)
BMI, kg/m2 (SD)	31.7 (5.7)	31.5 (6.0)	28.3 (5.2)	28.9 (4.5)	29.3 (4.6)	28.4 (4.7)	24.7 (3.7)	26.8 (3.6)	28.0 (4.8)	25.7 (3.8)	27.7 (3.9)	27.0 (4.1)
Mean SBP, mmHg (SD)	155 (17)	136 (13)	145 (22)	151 (15)	151 (14)	170 (14)	173 (9)	133 (17)	129 (17)	147 (19)	131 (19)	174 (10)
Mean DBP, mmHg (SD)	98 (7)	84 (3)	81 (11)	88 (8)	88 (8)	105 (3)	91 (9)	79 (10)	79 (9)	86 (11)	79 (10)	85 (6)
Mean total cholesterol level mmol/L (SD)	—	—	5.2 (1.2)	5.4 (1.0)	5.5 (1.0)	6.1 (1.1)	5.3 (1.1)	6.1 (1.1)	5.6 (1.0)	—	5.5 (0.8)	6 (1.2)
Mean HDL cholesterol level mmol/L (SD)	—	—	1.3 (0.4)	1.2 (0.3)	1.2 (0.3)	—	1.4 (0.4)	4.1 (0.9)	1.2 (0.3)	—	1.0 (0.2)	1.4 (0.5)
Previous antihypertensive treatment, n (%)	209 (44)	149 (31)	7,651 (69)	453 (100)	454 (100)	9,868 (53)	2,017 (54)	337 (55)	550 (67)	3,051 (50)	136 (57)	2,175 (47)
Smokers, n (%)	65 (14)	63 (13)	1,681 (15)	63 (14)	52 (11)	2,979 (16)	246 (7)	100 (16)	203 (25)	1,274 (21)	36 (15)	338 (7)
Diabetes mellitus, *n* (%)	470 (100)	479 (100)	11,133 (100)	453 (100)	454 (100)	1,501 (8)	369 (10)	51 (8)	98 (12)	759 (12)	23 (10)	483 (10)
Previous cardiovascular disease, n (%)	50 (11)	39 (8)	3,588 (32)	0 (0)	0 (0)	313 (2)	358 (10)	457 (74)	822 (100)	6,074 (100)	218 (91)	282 (6)
10-y total CVD risk, % (SD)	—	—	21 (12)	22 (11)	23 (11)	—	26 (13)	4 (3)	10 (7)	—	12 (8)	19 (11)
10-y CHD risk, % (SD)	—	—	15 (9)	15 (8)	15 (8)	—	17 (10)	3 (2)	7 (5)	—	9 (5)	12 (8)

10-y total CVD and CHD risks determined using the Framingham Heart Study equations. To convert from mmol/L to mg/dL for total cholesterol and HDL cholesterol, divide by 0.0259.

**Abbreviations:** ABCD, Appropriate Blood Pressure Control in Diabetics; ACEI, angiotensin-converting enzyme inhibitor; ADVANCE, Action in Diabetes and Vascular Disease: Preterax and Diamicron MR Controlled Evaluation; BENEDICT, Bergamo Nephrologic Diabetes Complications Trial; BMI, body mass index; CHD, coronary heart disease; CVD, cardiovascular disease; DBP, diastolic blood pressure; HDL, high-density lipoprotein; HOT, Hypertension Optimal Treatment; HYVET, Hypertension in the Very Elderly Trial; PART2, Prevention of Atherosclerosis with Ramipril; PREVENT, Prospective Randomized Evaluation of the Vascular Effects of Norvasc Trial; PROGRESS, Perindopril Protection Against Recurrent Stroke Study; SBP, systolic blood pressure; SCAT, Simvastatin/Enalapril Coronary Atherosclerosis Trial; SYST-EUR, Systolic Hypertension in Europe.

### Cardiovascular events avoided with treatment

**Fig 1:**
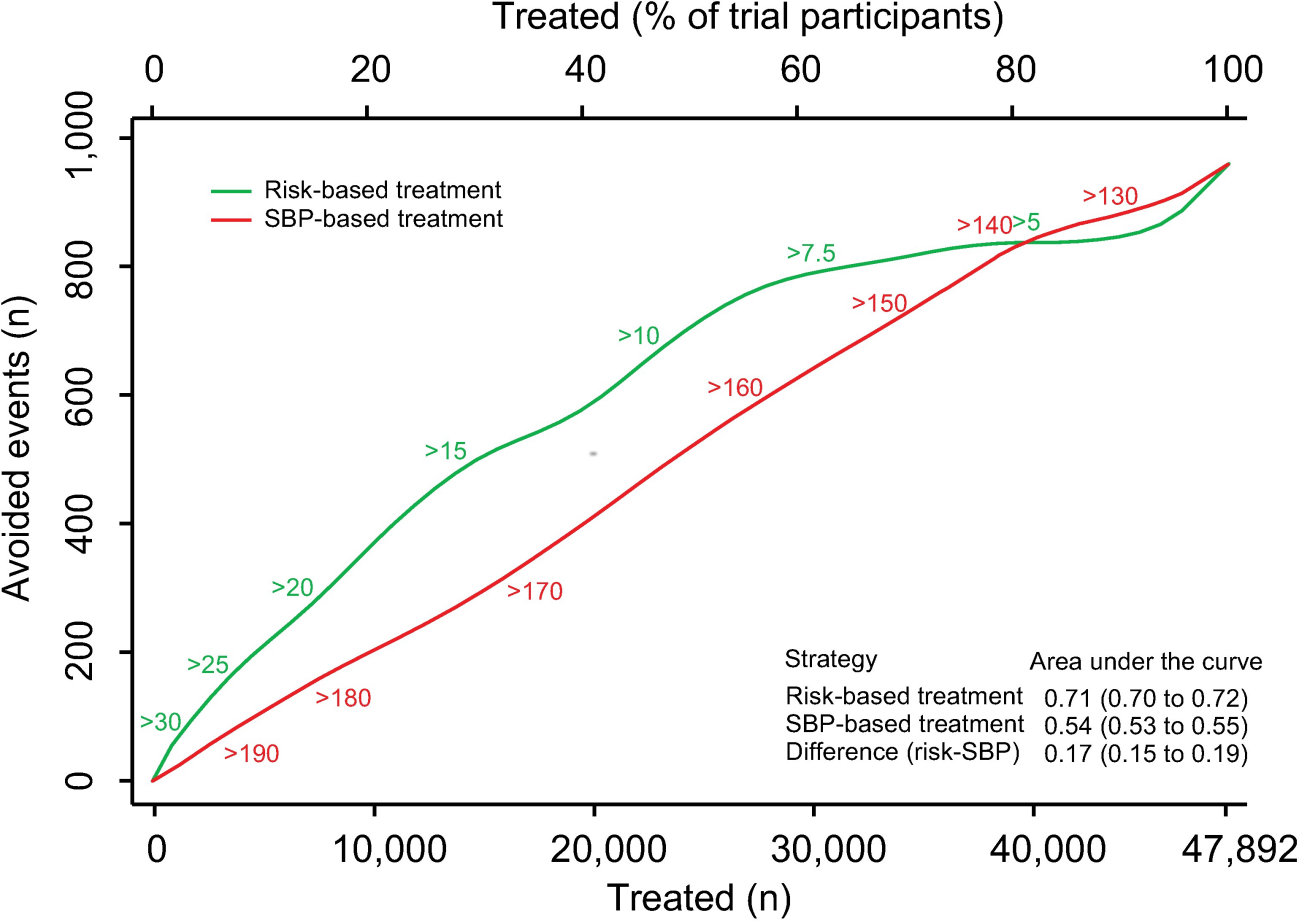
Effects of CVD risk and SBP treatment strategies on absolute number of cardiovascular events prevented over 5 y. Expected cardiovascular events avoided over 5 y of treatment as a function of number of persons and proportion of sample treated using a CVD risk strategy (in green) and an SBP strategy (in red). Numbers associated with each curve represent the specific CVD risk level (percentage 5-y CVD risk) or SBP (mmHg) at the treatment threshold. Areas under the curve are expressed as the ratio of the obtained area to the maximum possible area (maximum number of cardiovascular events avoided multiplied by the maximum number of participants treated) with bias-corrected 95% bootstrap CIs from 10,000 repetitions in parentheses. Larger areas represent more events avoided per persons treated. CI, confidence interval, CVD, cardiovascular disease; SBP, systolic blood pressure.

There were 3,566 (7.5%) participants who experienced an incident CVD event during a median follow-up of 4.0 y (IQR 1.0, S2 Table). We estimated the number of CVD events avoided over 5 y per person treated according to a CVD risk strategy compared with an SBP strategy (Fig 1). The CVD risk strategy would result in a greater number of events avoided per person treated compared with the SBP strategy. Similarly, for a given number of cardiovascular events avoided, a smaller proportion of the sample is treated using a CVD risk strategy compared with the SBP strategy. Areas under the curve comparing the two treatment strategies throughout the range of possible thresholds for CVD risk and SBP demonstrated that, on average, a greater number of CVD events would be avoided for a given number of persons treated with the CVD risk strategy compared with the SBP strategy (area under the curve 0.71 [95% CI 0.70–0.72] for the CVD risk strategy versus 0.54 [95% CI 0.53–0.55] for the SBP strategy) (Fig 1).

**Fig 2:**
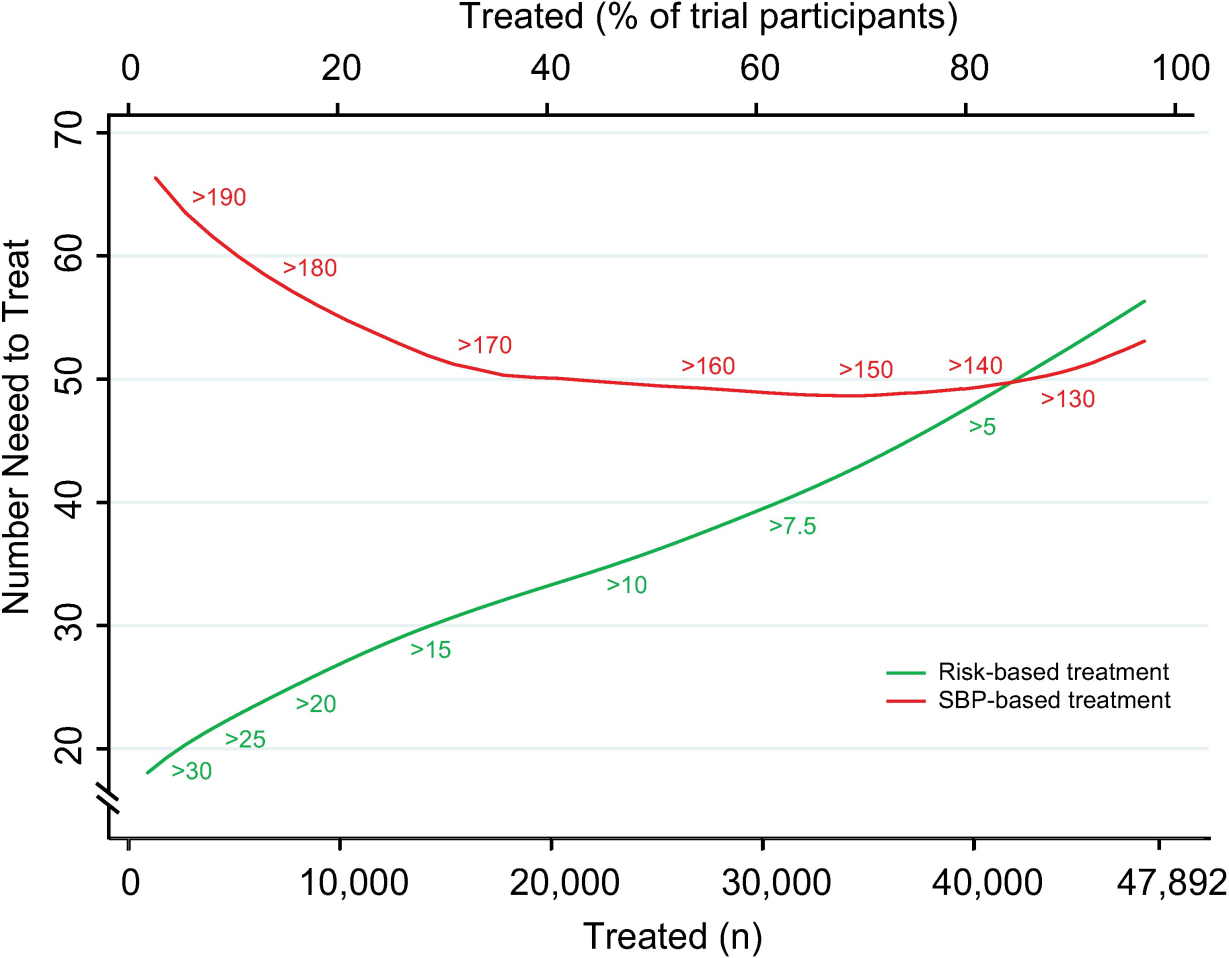
Numbers needed to treat for 5 y to avoid 1 cardiovascular event according to CVD risk and SBP treatment strategies. Numbers needed to treat for 5 y to avoid 1 cardiovascular event as a function of number of persons and proportion of sample treated using a CVD risk strategy (in green) and an SBP strategy (in red). Numbers associated with each curve represent the specific CVD risk level (percentage 5-y CVD risk) or SBP (mmHg) at the treatment threshold. CVD, cardiovascular disease; SBP, systolic blood pressure.

The number needed to treat for 5 y to prevent 1 CVD event was lower with the CVD risk strategy across a broad range of thresholds until overlapping with the SBP strategy at the 80th percentile treatment rate (Fig 2). Hence, the CVD risk strategy was superior to the SBP strategy in terms of identifying the persons with the highest absolute treatment benefit across a broad range of plausible treatment thresholds.

More specifically, we selected three commonly proposed SBP treatment thresholds for direct comparison with the CVD risk strategy (Tables 3 and 4). Compared with treatment at an SBP threshold of 150 mmHg, a CVD risk threshold would require the treatment of 29% (95% CI 26%–31%) fewer individuals to prevent the same number of CVD events (Table 3). Alternatively, it could prevent 16% (95% CI 14%–18%) more CVD events for the same number of persons treated (Table 4). Similarly, compared with an SBP threshold of 160 mmHg, using a CVD risk threshold would require treatment of 35% (95% CI 50%–24%) fewer persons to prevent the same number of CVD events, or it could prevent 38% (95% CI 29%–40%) more CVD events for the same number of treated persons. Results at an SBP threshold of 140 mmHg were imprecise due to the nature of our comparisons. At this threshold, the CVD risk strategy would prevent 3% (95% CI 1.5%–5%) more CVD events for the same number of persons treated and require 3.8% fewer (12.5% fewer to 7.2% more) persons to be treated for the same number of events prevented, although this result was not statistically significant.

**Table 3:** Persons needed to treat to avoid the same number of cardiovascular events over 5 y using selected SBP thresholds and corresponding CVD risk thresholds. The BPLTTC (*n* = 47,872).

		SBP threshold		
		140 mmHg	150 mmHg	160 mmHg
Persons and proportions treated using an SBP threshold	*n*	39,231 (39,231 to 39,344)	33,891 (33,551 to 34,460)	27,039 (26,245 to 27,882)
	percentage	81.9 (81.9 to 82.2)	70.8 (70.1 to 72.0)	56.5 (54.8 to 58.2)
Persons and proportions treated using a CVD risk threshold selected to avoid the same number of cardiovascular events	*n*	37,730 (34,343 to 42,073)	24,225 (23,743 to 25,660)	17,484 (14,108 to 21,333)
	percentage	78.8 (71.7 to 87.9)	50.6 (49.6 to 53.6)	36.5 (29.5 to 44.6)
Difference in persons and relative difference in proportions treated using SBP and CVD risk thresholds selected to avoid the same number of cardiovascular events	*n*	−1,501 (−4,888 to +2,842)	−9,667 (−10,717 to −8,504)	−9,556 (−13,095 to −6,603)
	percentage	−3.8 (−12.5 to +7.2)	−28.5 (−31.1 to −25.6)	−35.3 (−49.9 to −24.2)

Estimates with 10,000 repetitions bias-corrected bootstrap with 95% CIs in parentheses. Negative sign represents smaller proportion or lesser number. Positive sign represents bigger proportion or greater number.

**Abbreviations:** BPLTTC, Blood Pressure Lowering Treatment Trialists’ Collaboration; CI, confidence interval; CVD, cardiovascular disease; SBP, systolic blood pressure.

**Table 4:** Cardiovascular events avoided over 5 y for the same number of persons treated using selected SBP thresholds and corresponding CVD risk thresholds. The BPLTTC (*n* = 47,872).

		SBP threshold		
		140 mmHg	150 mmHg	160 mmHg
		*n* = 39,231	*n* = 33,891	*n* = 27,039
CVD events avoided using an SBP threshold	*n*	821 (819 to 821)	698 (692 to 716)	557 (544 to 599)
CVD events avoided using a CVD risk threshold selected to achieve the same number of persons treated	*n*	847 (833 to 860)	809 (795 to 822)	767 (755 to 776)
Difference and relative difference in CVD events avoided using SBP and CVD risk thresholds selected to achieve the same number of persons treated	*n*	+26 (+12 to +41)	+111 (+97 to +127)	+210 (175 to 222)
	percentage	+3.1 (+1.5 to +5.0)	+15.8 (+13.7 to +18.3)	+37.6 (+28.8 to +40.2)

Estimates with 10,000 repetitions bias-corrected bootstrap with 95% CIs in parentheses. Negative sign represents smaller proportion or lesser number. Positive sign represents greater number.

**Abbreviations:** BPLTTC, Blood Pressure Lowering Treatment Trialists’ Collaboration; CI, confidence interval; CVD, cardiovascular disease; SBP, systolic blood pressure.

### Secondary analyses

**Fig 3:**
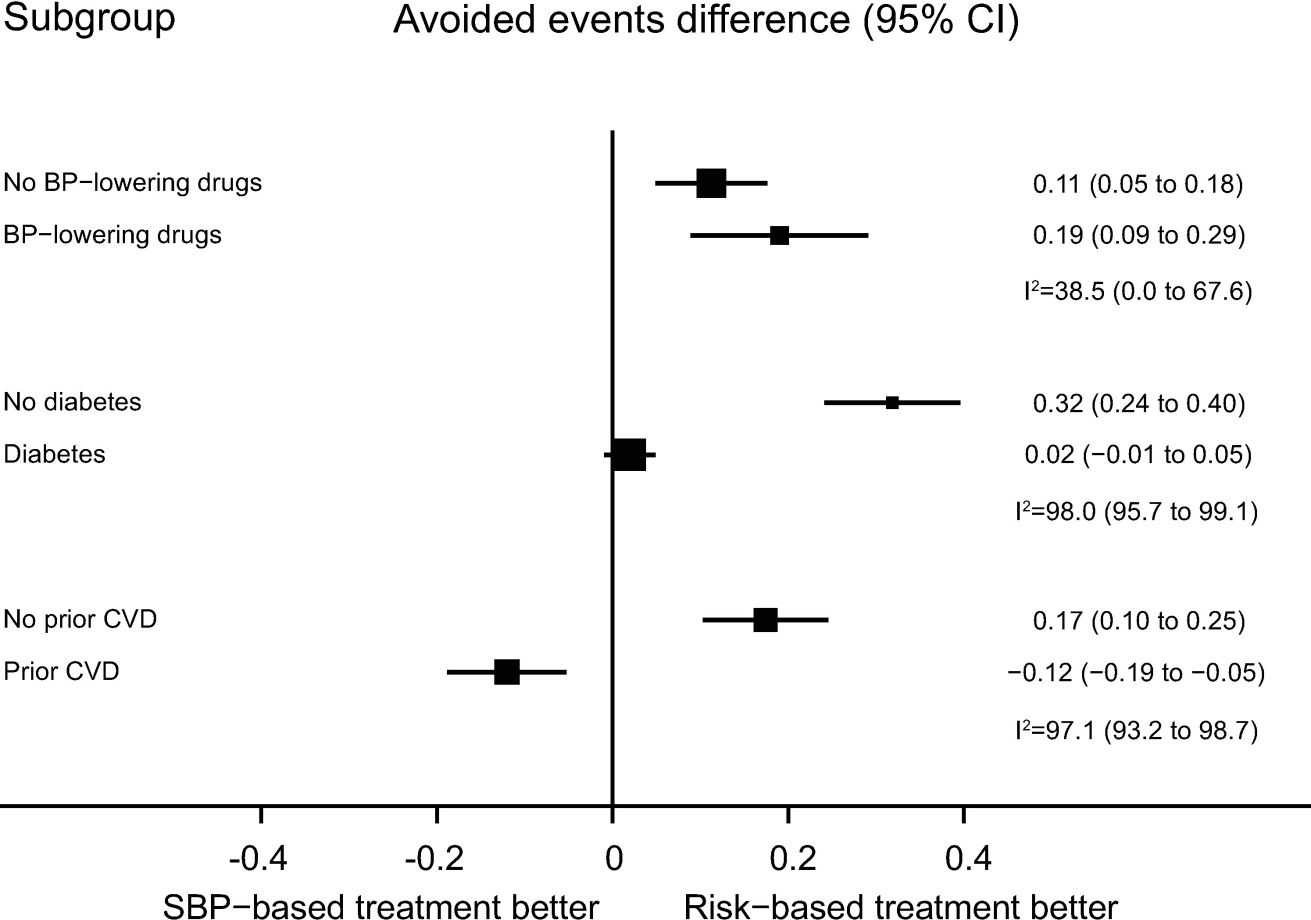
Performance of CVD risk and SBP treatment strategies in subgroups defined at baseline. Estimates represent differences in areas under the curve between CVD risk and SBP treatment strategies for the number of cardiovascular events avoided per persons treated in each subgroup, defined at baseline. Bias-corrected 95% bootstrap CIs from 10,000 repetitions in parentheses. Heterogeneity for the dataset determined for meta-analyses of two complementary strata at a time was assessed using I^2^ and corresponding 95% CIs. BP, blood pressure; CI, confidence interval, CVD, cardiovascular disease; SBP, systolic blood pressure.

Results were similar in subgroups with and without prior blood pressure-lowering medication use, without diabetes mellitus, and without prevalent CVD (Fig 3). For those with baseline diabetes mellitus, the CVD risk strategy did not appear to be superior, while for those with prior CVD, the SBP strategy appeared to be best (Fig 3).

**Fig 4:**
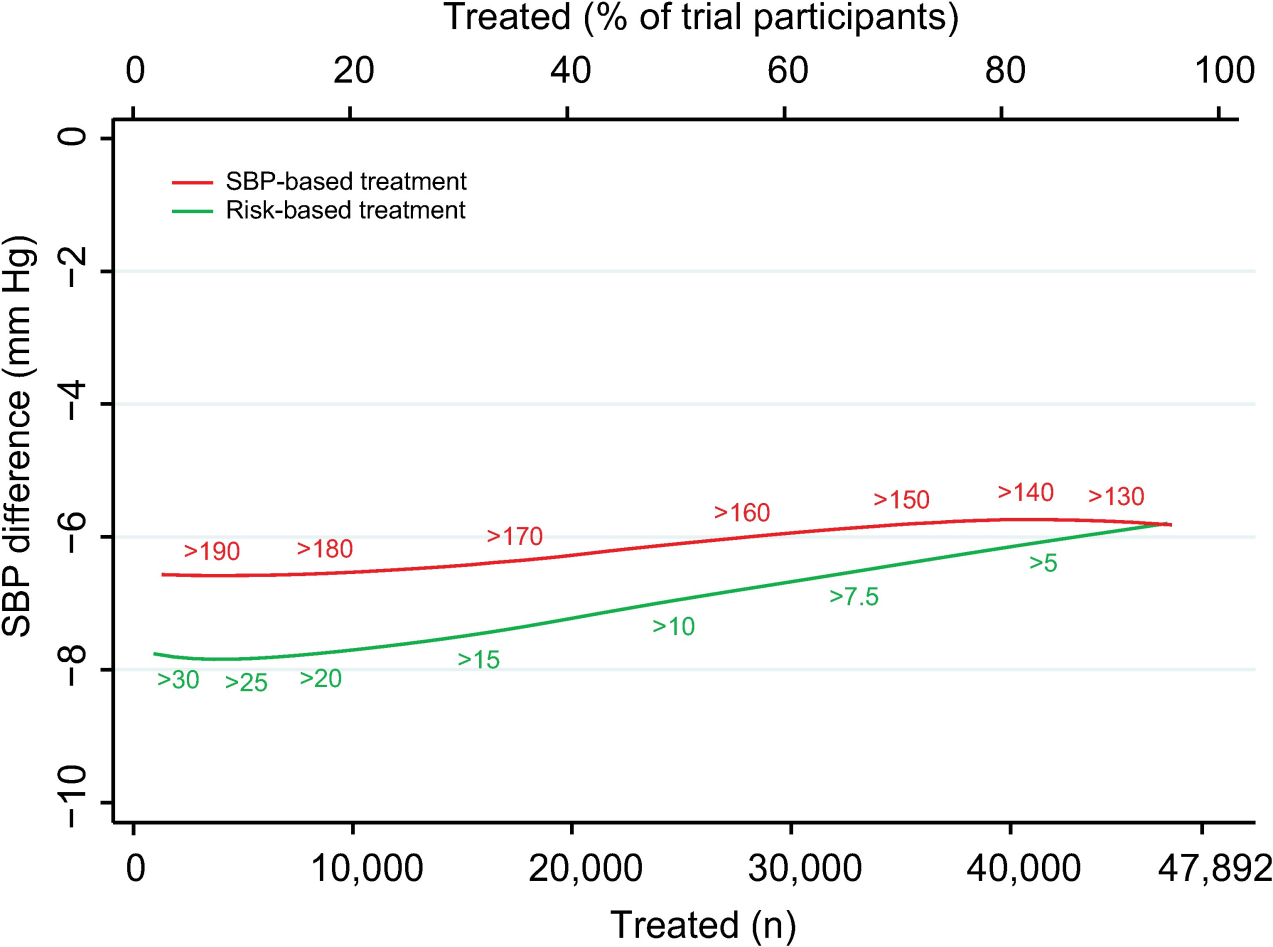
Achieved SBP reduction according to CVD risk and SBP treatment strategies. Achieved SBP reduction as a function of number of persons and proportion of sample treated using a CVD risk strategy (in green) and an SBP strategy (in red). Numbers associated with each curve represent the specific CVD risk level (percentage 5-y CVD risk) or SBP (mmHg) at the treatment threshold. CVD, cardiovascular disease; SBP, systolic blood pressure.

**Fig 5:**
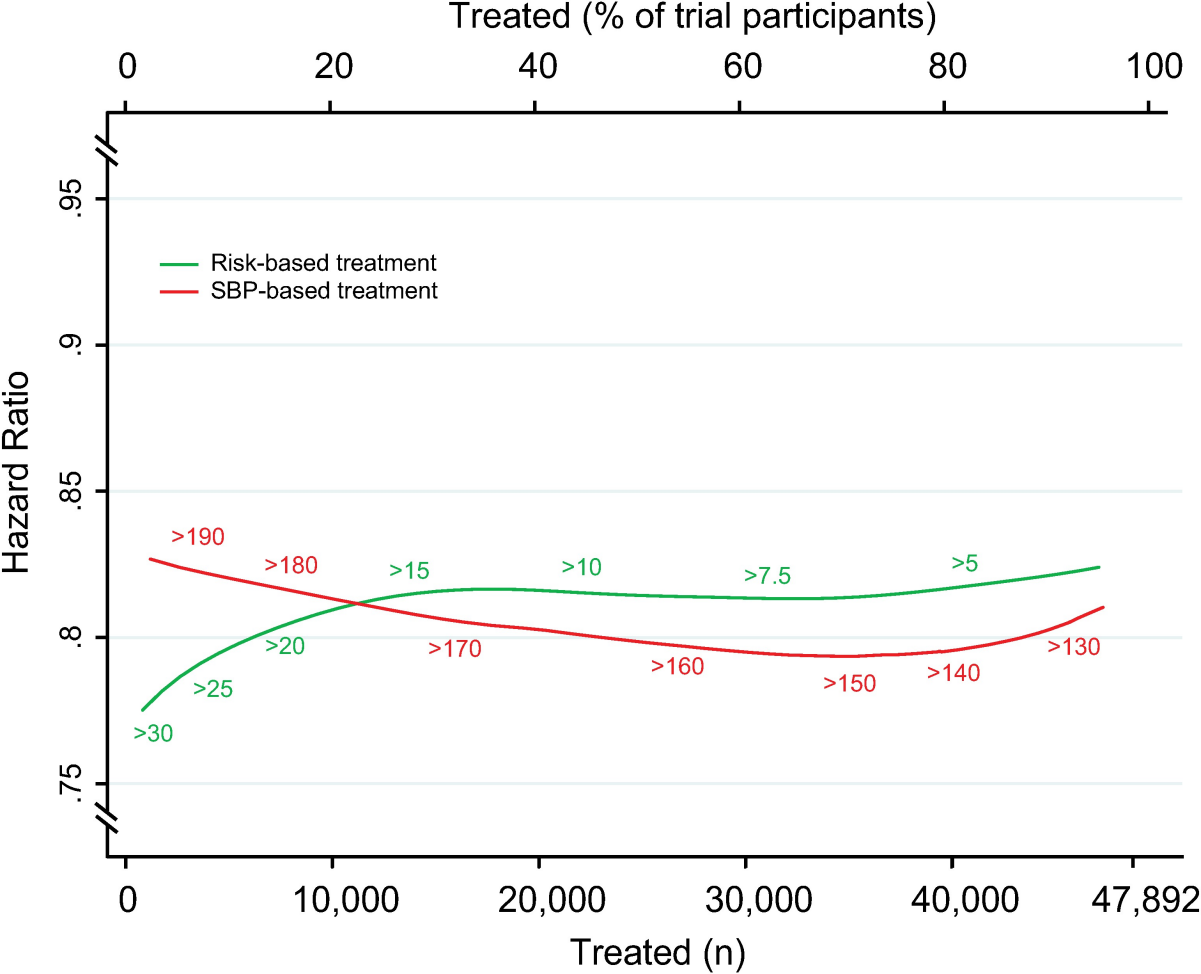
Relative risk reductions by blood pressure-lowering treatment, according to CVD risk and SBP treatment strategies. Hazard ratios of major cardiovascular events by blood pressure-lowering treatment, as a function of number of persons and proportion of sample treated using a CVD risk strategy (in green) and an SBP strategy (in red). Numbers associated with each curve represent the specific CVD risk level (percentage 5-y CVD risk) or SBP (mmHg) at the treatment threshold. Hazard ratios determined from persons above each treatment threshold. CVD, cardiovascular disease; SBP, systolic blood pressure.

Mean achieved SBP reductions and observed relative risk reductions for both the SBP strategy and CVD risk strategy were similar across the range of possible CVD risk and SBP thresholds (Fig 4 and Fig 5).

Results were similar for analyses standardized to a 5-mmHg SBP reduction (S2 Fig and S3 Fig), but differences were smaller. Furthermore, analyses comparing the two treatment strategies with an age-based treatment strategy confirmed that the CVD risk strategy remained superior in terms of events avoided per person treated compared with the SBP strategy (difference in areas under the curves 0.17 [95% CI 0.15–0.19]) and age-based strategy (difference in areas under the curves 0.13 [95% CI 0.09–0.14]) (S4 Fig).

For analyses using the Framingham total CVD risk equation, a greater number of CVD events would be avoided for a given number of persons treated with the CVD risk strategy compared with the SBP strategy (area under the curve 0.66 [95% CI 0.65–0.72] for CVD risk strategy versus 0.59 [95% CI 0.57–0.61] for the SBP strategy), although differences were smaller than in analyses using the internally derived risk prediction equation.

## Discussion

This analysis of nearly 50,000 persons studied in clinical trials demonstrated that a blood pressure-lowering treatment strategy based on predicted CVD risk could prevent more events for the same number of persons treated compared with a strategy based on SBP levels. The benefit of the CVD risk strategy was particularly evident at higher SBP thresholds and for persons without prevalent CVD or diabetes mellitus.

The superiority of the CVD risk strategy relative to the SBP strategy can be explained by recent lessons from blood pressure epidemiology. First, the relative treatment benefit from blood pressure-lowering medications is fairly consistent across different blood pressure levels, including nonhypertensive levels [6,7]. Second, the absolute risk of an individual at a given blood pressure level can vary as much as 20-fold based on the presence of other vascular risk factors such as age, sex, dyslipidemia, and diabetes mellitus [5,29]. Third, a previous meta-analysis of individual participant data from the BPLTTC has shown that the relative benefit from blood pressure-lowering treatment is similar across risk strata and that, therefore, the absolute benefit from blood pressure-lowering treatment is greater in those with higher risk [11]. Therefore, the expected absolute risk reduction achieved with blood pressure-lowering treatment is better determined by the combination of risk factors contributing to CVD risk rather than an isolated blood pressure level. In the present study, we quantified the benefits of such a risk-based strategy and determined which groups of patients might experience those benefits.

Indirect comparisons of CVD risk and SBP treatment strategies have previously been performed using observational cohorts and modeled treatment effects. These analyses suggest that treatment strategies based on absolute risk could prevent more cardiovascular events, save more quality-adjusted life-years, use fewer medications, and lower overall costs compared with treatment based on blood pressure level [12,13,30,31]. Our results confirm, quantify, and extend these findings in a large group of persons who actually received blood pressure-lowering medications in randomized clinical trials across a broad range of CVD risk and SBP levels.

It should be noted that this is a proof-of-principle study. The results of the two strategies will, by design, appear to converge at the upper and lower ends of the CVD risk and SBP distributions that are determined by the composition of this sample and findings at the ends of the distributions are uncertain. Therefore, although the results of the present study illustrate the principle that a CVD risk strategy outperforms an SBP strategy over a broad range of SBPs and CVD risks, there is a need for further studies elucidating the differences between these two strategies at the lower ends of the blood pressure and CVD risk distributions, particularly because these are values that are taken into consideration when discussing thresholds for therapeutic intervention. The Systolic Blood Pressure Intervention Trial (SPRINT) and the Heart Outcomes Prevention Evaluation-3 (HOPE-3) study provide data that complement our analyses and support these results [32,33]. In SPRINT, blood pressure reduction in a high-risk group (annual event rate of 2.2%/y) below conventional targets led to a 25% reduction in cardiovascular events and a 27% reduction in all-cause deaths [32]. In contrast, HOPE-3 did not demonstrate a cardiovascular benefit from blood pressure reduction in a lower-risk group (annual event rate 0.8%/y) where the majority of participants had SBP below conventional targets [33].

In subgroup analyses, we found no evidence of a benefit for a CVD risk strategy compared with SBP strategy in patients with diabetes mellitus or existing CVD. This may reflect that “high risk” is already captured in these groups, independent of the predicted CVD risk estimate. In addition, the relatively weaker performance of CVD risk prediction among persons with existing CVD could also be explained by phenomena like index event bias [34] or observations that consequences of a cardiovascular event are often stronger predictors of a subsequent event than traditional risk factors [35–37]. Given the more uniformly high-risk status of persons with prevalent CVD or diabetes mellitus, a “treat-all” strategy may be better than selective treatment based on predicted CVD risk or blood pressure level. Thus, utilizing predicted CVD risk to guide treatment decisions may be best suited in primary prevention for individuals without diabetes mellitus or CVD where baseline risks and expected benefits from blood pressure-lowering treatment are more heterogeneous. This study, therefore, provides clinical guidance for treatment decisions in a broad segment of the general population.

### Clinical implications

Our results support the use of absolute risk assessment in guiding blood pressure-lowering treatment decisions. Although risk-based treatment has been a cornerstone of cholesterol management [9,10], blood pressure treatment guidelines like those from the US have historically emphasized blood pressure thresholds and targets [1,38]. The present study challenges this paradigm and instead highlights the merits of using predicted CVD risk to guide intensity of blood pressure-lowering with medications, a framework recently embraced by the 2017 American College of Cardiology (ACC)/American Heart Association (AHA) Hypertension guidelines [39]. The limited trial experience with treatment to SBP below 120 mmHg necessarily limits the scope of an entirely risk-based treatment strategy. Nevertheless, our results support the principle that treatment decisions that are based on absolute CVD risk compared with blood pressure alone are superior for identifying persons with the highest expected benefit from blood pressure-lowering treatment, especially in primary prevention. This assessment can form the basis for a shared clinician–patient discussion to contextualize expected benefits and harms of blood pressure treatment with individual values and preferences to personalize treatment decisions.

### Strengths and limitations

A key strength of this analysis is the use of a high-quality dataset of persons who were actually treated with blood pressure-lowering medications as part of a randomized clinical trial with rigorous follow-up and adjudicated outcomes (S5 Fig). Furthermore, our calculation of relative risk reductions from blood pressure-lowering treatment at each potential threshold preserves the randomized structure of the trials and maximizes the information within this dataset. There are, however, important limitations to acknowledge in this analysis. First, most trial participants were hypertensive at baseline, were receiving background blood pressure-lowering therapy, and had an estimated 5-y CVD risk greater than 5%. Therefore, the majority of the difference between the two strategies was seen among participants with SBP 150 to 170 mmHg or 5-y CVD risk 7.5% to 15%. As such, we were unable to detect a difference between the two treatment strategies at a 5-y CVD risk level of less than 5% or at an SBP threshold of 140 mmHg. The inability of this method to provide information at the ends of the risk and blood pressure distributions motivates future studies in other samples that include individuals with lower blood pressures or risk. Second, trials were of relatively short duration, and therefore, these results do not account for the potential long-term benefits (or harms) of sustained blood pressure management over the course of a lifetime [40]. Third, the utility of a CVD risk strategy depends on the performance of the prediction algorithm employed. For this study, we chose a previously validated, internally derived prediction algorithm to optimize model performance. Thus, these results have high internal but unknown external validity. To account for this limitation, we performed sensitivity analyses using the Framingham total CVD risk equation. The results, while qualitatively similar, had a lower area under the curve, suggesting worse performance of the CVD risk strategy using an externally derived prediction algorithm. It should be noted that modifying the used risk equation to prediction of 10- instead of 5-y risks would yield the same ranking of participants and hence produce the same relative results. Finally, this is a post-hoc analysis of data from clinical trials that had different objectives and entry criteria and with numerical results driven by the composition of the study sample. Therefore, prospective validation in a new trial would be the ideal way to confirm our findings, but given the large number of participants required, such a trial is unlikely to be performed. These limits notwithstanding, these results should serve as a proof of principle of the relative merits of a CVD risk blood pressure treatment strategy in a high-quality dataset of persons treated with blood pressure-lowering medications.

### Conclusion

In conclusion, this individual participant data analysis of blood pressure-lowering trial participants supports the principle that a treatment strategy based on predicted CVD risk, compared with one based on SBP levels, would result in prevention of more cardiovascular events for the same number of treated persons across a wide range of potential treatment thresholds. These results support use of cardiovascular risk assessment to guide blood pressure-lowering treatment decision-making in moderate- to high-risk individuals, especially in primary prevention settings.

## Supporting information

S1 Table Mean blood pressure reductions in included trials. (DOCX)

S2 Table Number of events by trial included in analysis. (DOCX)

S1 Fig Observed versus expected 5-y risks of cardiovascular events in prespecified subgroups. Calibration of the internally derived CVD risk prediction equation in the prespecified subgroups. CVD, cardiovascular disease.
(PDF)

S2 Fig Effects of CVD risk and SBP treatment strategies on absolute number of cardiovascular events prevented, standardized to a 5-mmHg SBP reduction. Expected cardiovascular events avoided over 5 y as a function of number of persons and proportion of sample treated using a CVD risk strategy (in green) and an SBP strategy (in red), standardized to a 5-mmHg SBP reduction. Numbers associated with each curve represent the specific CVD risk level (percentage 5-y CVD risk) or SBP (mmHg) at the treatment threshold. Areas under the curve are expressed as the ratio of the obtained area to the maximum possible area (maximum number of cardiovascular events avoided multiplied by the maximum number of persons treated) with bias-corrected 95% bootstrap CIs from 10,000 repetitions in parentheses. Larger areas represent more avoidable events avoided per persons treated. CI, confidence interval; CVD, cardiovascular disease; SBP, systolic blood pressure.
(PDF)

S3 Fig Numbers needed to treat for 5 y to avoid 1 cardiovascular event according to CVD risk and SBP treatment strategies, standardized to a 5-mmHg SBP reduction. Numbers needed to treat for 5 y to avoid 1 cardiovascular event as a function of number of persons and proportion of sample treated using a CVD risk strategy (in green) and an SBP strategy (in red), standardized to a 5-mmHg SBP reduction. Numbers associated with each curve represent the specific CVD risk level (percentage 5-y CVD risk) or SBP (mmHg) at the treatment threshold. CVD, cardiovascular disease; SBP, systolic blood pressure.
(PDF)

S4 Fig Effects of CVD risk–, SBP-, and age-based treatment strategies on absolute number of cardiovascular events prevented. Expected cardiovascular events avoided over 5 y as a function of number of persons and proportion of sample treated using a CVD risk strategy (in green), an SBP strategy (in red), and an age-based strategy (in blue). Numbers associated with each curve represent the specific CVD risk (percentage 5-y CVD risk), SBP (mmHg), or age (y) level at that treatment threshold. CVD, cardiovascular disease; SBP, systolic blood pressure.
(PDF)

S5 Fig Flow chart. (PDF)

## Abbreviations

ABCD: Appropriate Blood Pressure Control in Diabetics
ACC: American College of Cardiology
ACE: angiotensin-converting enzyme
ACEI: angiotensin-converting enzyme inhibitor
ADVANCE: Action in Diabetes and Vascular Disease: Preterax and Diamicron MR Controlled Evaluation
AHA: American Heart Association
BENEDICT: Bergamo Nephrologic Diabetes Complications Trial
BPLTTC: Blood Pressure Lowering Treatment Trialists’ Collaboration
CI: confidence interval
CVD: cardiovascular disease
DBP: diastolic blood pressure
HDL: high-density lipoprotein
HOPE-3: Heart Outcomes Prevention Evaluation-3
HOT: Hypertension Optimal Treatment
HYVET: Hypertension in the Very Elderly Trial
PART2: Prevention of Atherosclerosis with Ramipril
PREVENT: Prospective Randomized Evaluation of the Vascular Effects of Norvasc Trial
PROGRESS: Perindopril Protection Against Recurrent Stroke Study
SBP: systolic blood pressure
SCAT: Simvastatin/Enalapril Coronary Atherosclerosis Trial
SPRINT: Systolic Blood Pressure Intervention Trial
SYST-EUR: Systolic Hypertension in Europe
