# Supplementary material for: Blood pressure-lowering treatment strategies based on cardiovascular risk versus blood pressure: A meta-analysis of individual participant data
Source: PLoS Med. 2018 Mar 20;15(3):e1002538. doi: 10.1371/journal.pmed.1002538 (PMC5860698; doi:10.1371/journal.pmed.1002538)
Supplement: S1 Table — (DOCX) [file pmed.1002538.s001.docx]

**S1 Table. Mean blood pressure reductions in included trials**

| **Trial** | **Treatment regimen** | **Mean systolic blood pressure difference, mmHg (95% CI)** | **Mean diastolic blood pressure difference, mmHg (95% CI)** |
| --- | --- | --- | --- |
| ABCD_Hypertensive sample | More vs. less intense | -8.6 (-11.1 to -6.0) | -7.6 (-8.8 to -6.4) |
| ABCD_Normotensive sample | More vs. less intense | -6.7 (-8.5 to -4.9) | -5.7 (-6.5 to -4.9) |
| ADVANCE | Perindopril + indapamide vs. placebo | -5.5 (-6.1 to -4.9) | -2.2 (-2.5 to -1.9) |
| BENEDICT_ACEI | Trandolapril vs. placebo | -2.3 (-4.8 to -0.2) | -1.6 (-2.9 to -0.2) |
| BENEDICT_CCB | Verapamil vs. placebo | 1.8 (-0.7 to 4.3) | 0.4 (-1.1 to 1.8) |
| HOT | More vs. less intense | -2.8 (-3.2 to -2.4) | -3.0 (-3.2 to -2.8) |
| HYVET | Indapamide (+perindopril) vs. placebo | -9.7 (-10.6 to -8.9) | -4.0 (-4.6 to -3.5) |
| PART2 | Ramipril vs. placebo | -6.0 (-8.1 to -4.0) | -3.6 (-4.8 to -2.3) |
| PREVENT | Amlodipine vs. placebo | -5.3 (-7.2 to -3.4) | -3.2 (-4.2 to -2.2) |
| PROGRESS | Perindopril (+indapamide) vs. placebo | -9.0 (-9.8 to -8.2) | -3.9 (-4.4 to -3.5) |
| SCAT | Enalapril vs. placebo | -5.1 (-9.2 to -1.0) | -3.0 (-5.3 to -0.8) |
| SYST-EUR | Nifedipine vs. placebo | -10.1 (-10.8 to -9.4) | -3.9 (-4.2 to -3.5) |

*Trial Abbreviations:* ABCD = Appropriate Blood Pressure Control in Diabetics; ACEI = angiotensin-converting enzyme inhibitor; ADVANCE = Action in Diabetes and Vascular Disease: Preterax and Diamicron MR Controlled Evaluation; BENEDICT = Bergamo Nephrologic Diabetes Complications Trial; HOT = Hypertension Optimal Treatment; HYVET = Hypertension in the Very Elderly Trial; PART2 = Prevention of Atherosclerosis with Ramipril; PREVENT = Prospective Randomized Evaluation of the Vascular Effects of Norvasc Trial; PROGRESS = Perindopril Protection Against Recurrent Stroke Study; SCAT = Simvastatin/Enalapril Coronary Atherosclerosis Trial; SYST-EUR = Systolic Hypertension in Europe
