# Supplementary material for: Blood pressure-lowering treatment strategies based on cardiovascular risk versus blood pressure: A meta-analysis of individual participant data
Source: PLoS Med. 2018 Mar 20;15(3):e1002538. doi: 10.1371/journal.pmed.1002538 (PMC5860698; doi:10.1371/journal.pmed.1002538)
Supplement: S2 Table — (DOCX) [file pmed.1002538.s002.docx]

**S2 Table. Number of events by trial included in analysis**

| **Group** | **Participants, n** | **Total incident CVD, n** | **Stroke,**  **n** | **CHD,**  **n** | **CHF,**  **n** | **Death,**  **n** |
| --- | --- | --- | --- | --- | --- | --- |
| Per treatment allocation | | | | | | |
| Drug/More intense | 21,021 | 1,519 | 688 | 669 | 333 | 652 |
| Placebo/Less intense | 26,851 | 2,047 | 959 | 876 | 398 | 837 |
| Per trial | | | | | | |
| ABCD_H | 470 | 91 | 24 | 40 | 23 | 38 |
| ABCD_N | 479 | 75 | 16 | 34 | 23 | 38 |
| ADVANCE | 11,133 | 1,000 | 433 | 559 | 395 | 879 |
| BENEDICT_ACEI | 453 | 8 | 2 | 5 | 1 | 5 |
| BENEDICT_CCB | 454 | 7 | 2 | 4 | 1 | 4 |
| HOT | 18,776 | 682 | 293 | 355 | 22 | 589 |
| HYVET | 3,719 | 211 | 0 | 21 | 75 | 0 |
| PART2 | 617 | 81 | 11 | 59 | 17 | 41 |
| PREVENT | 822 | 53 | 9 | 39 | 6 | 14 |
| PROGRESS | 6,074 | 1,056 | 723 | 287 | 144 | 617 |
| SCAT | 240 | 25 | 9 | 10 | 4 | 9 |
| SYST-EUR | 4,635 | 277 | 125 | 132 | 20 | 274 |
| Total | 47,872 | 3,566 | 1,647 | 1,545 | 731 | 2,508 |

Some participants may have suffered from more than one type of outcome
