# Supplementary figures and images for: Blood pressure-lowering treatment strategies based on cardiovascular risk versus blood pressure: A meta-analysis of individual participant data
Source: PLoS Med. 2018 Mar 20;15(3):e1002538. doi: 10.1371/journal.pmed.1002538 (PMC5860698; doi:10.1371/journal.pmed.1002538)

**No diabetes**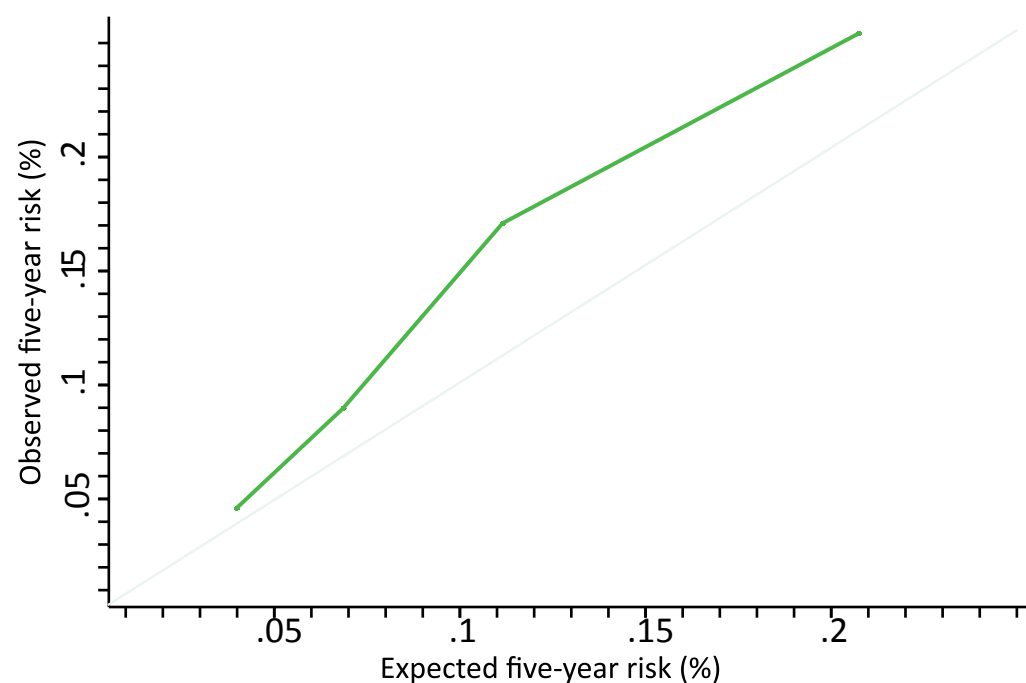**Diabetes**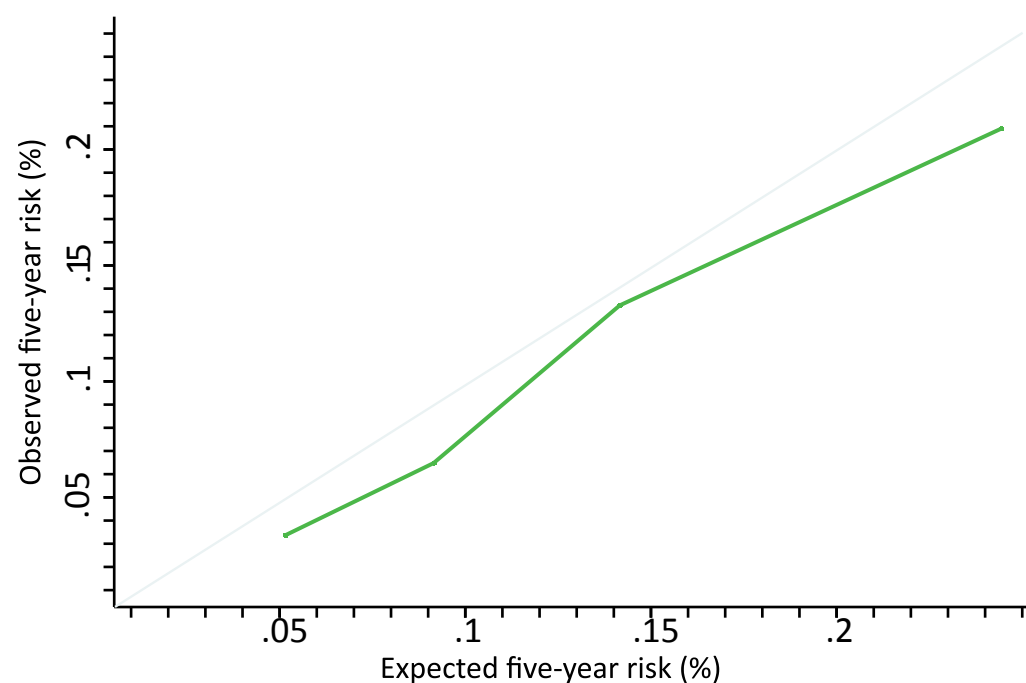**No BP-lowering drugs**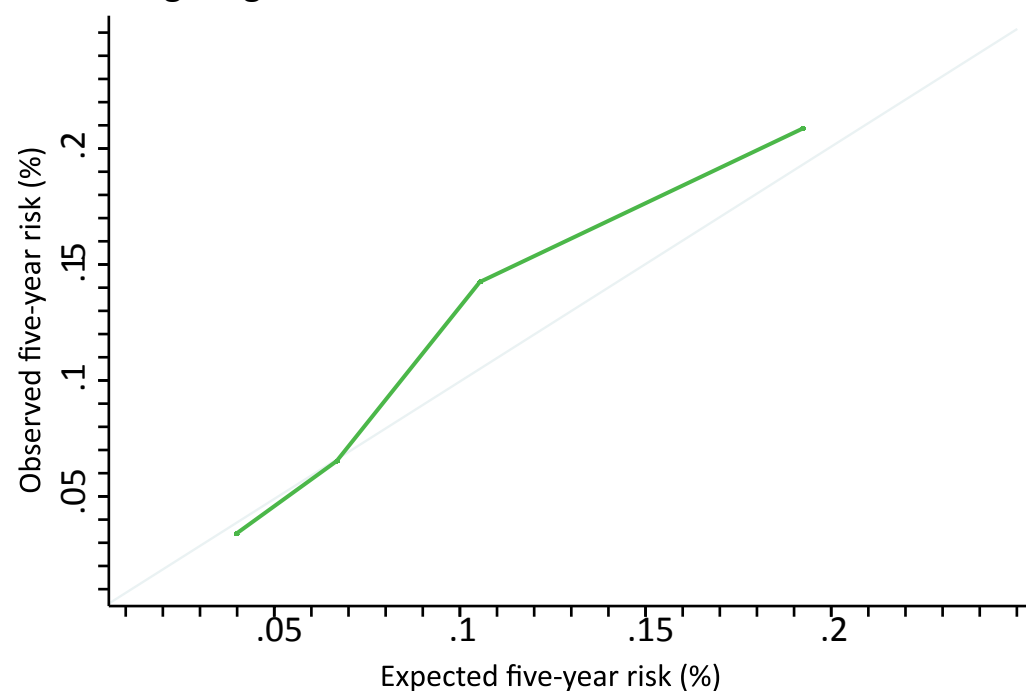**BP-lowering drugs**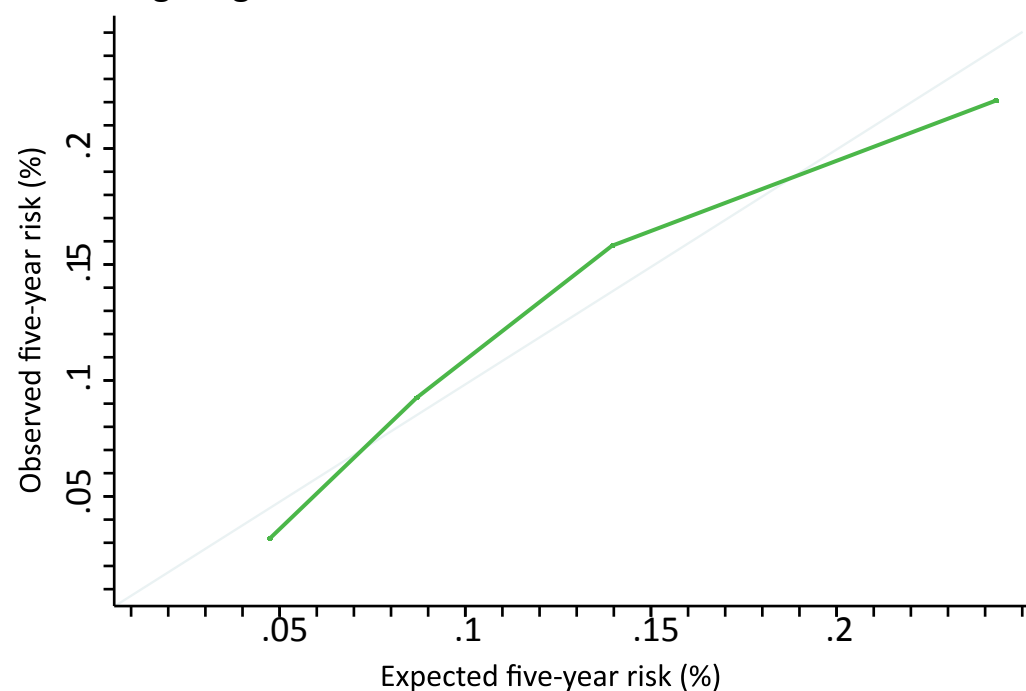**No prior CVD**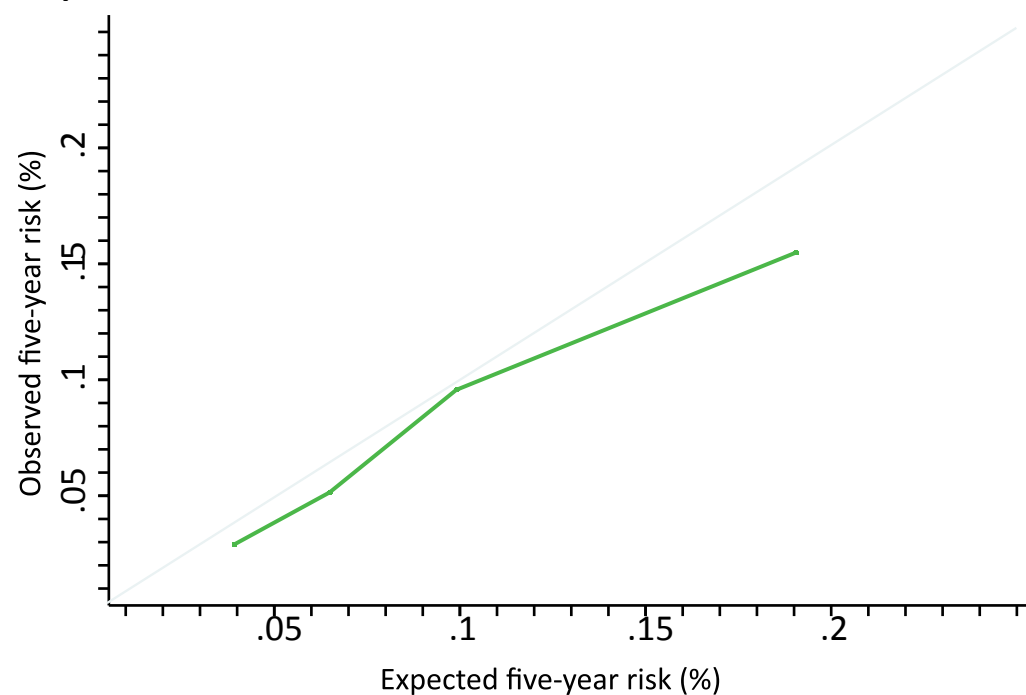**Prior CVD**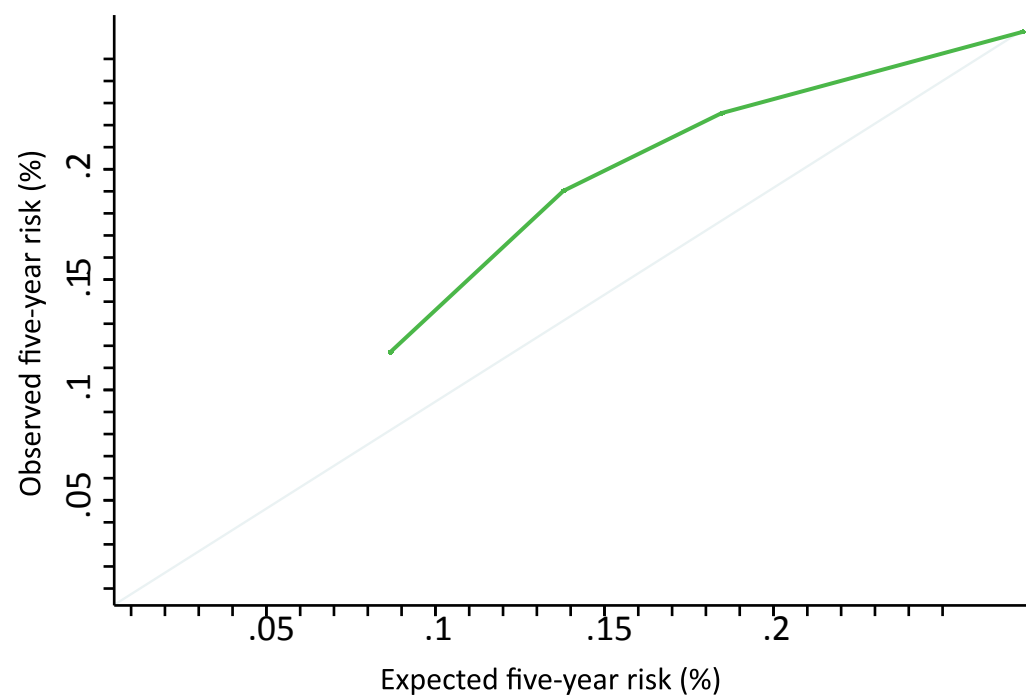

Supplement: S1 Fig — Calibration of the internally derived CVD risk prediction equation in the prespecified subgroups. CVD, cardiovascular disease. (PDF) [file pmed.1002538.s003.pdf]

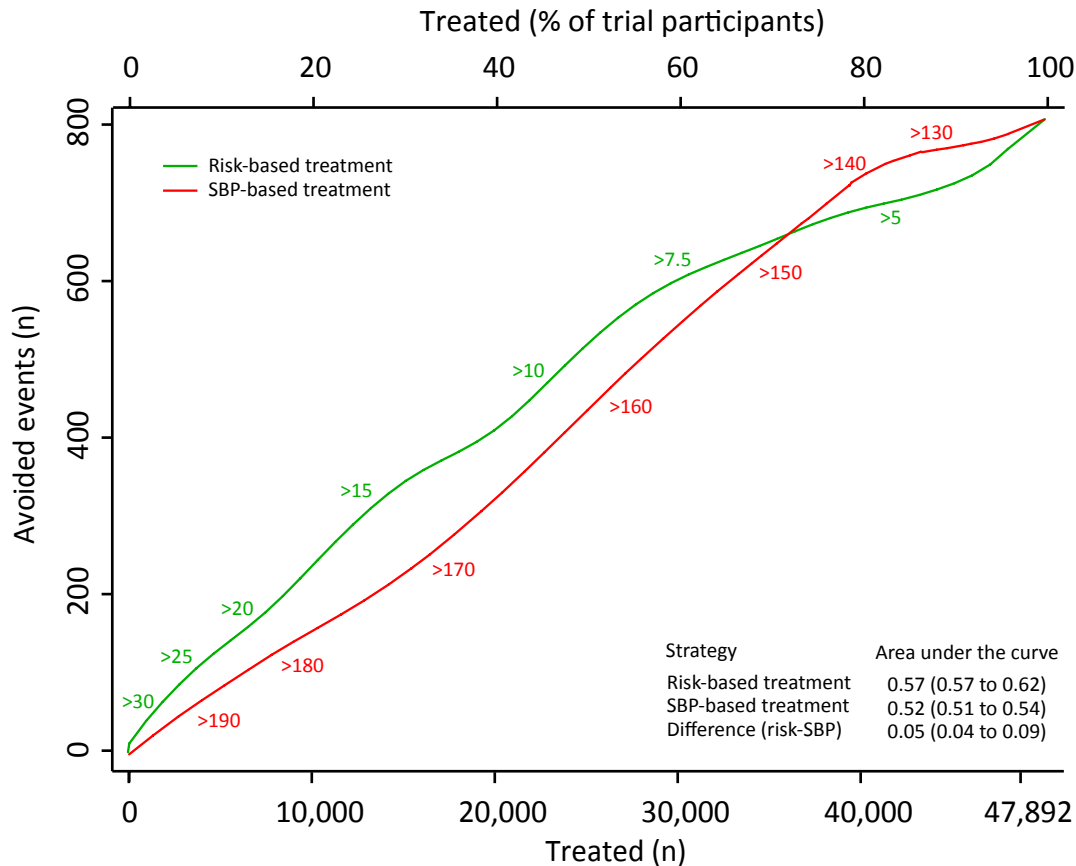

Supplement: S2 Fig — Expected cardiovascular events avoided over 5 y as a function of number of persons and proportion of sample treated using a CVD risk strategy (in green) and an SBP strategy (in red), standardized to a 5-mmHg SBP reduction. Numbers associated with each curve represent the specific CVD risk level (percentage 5-y CVD risk) or SBP (mmHg) at the treatment threshold. Areas under the curve are expressed as the ratio of the obtained area to the maximum possible area (maximum number of cardiovascular events avoided multiplied by the maximum number of persons treated) with bias-corrected 95% bootstrap CIs from 10,000 repetitions in parentheses. Larger areas represent more avoidable events avoided per persons treated. CI, confidence interval; CVD, cardiovascular disease; SBP, systolic blood pressure. (PDF) [file pmed.1002538.s004.pdf]

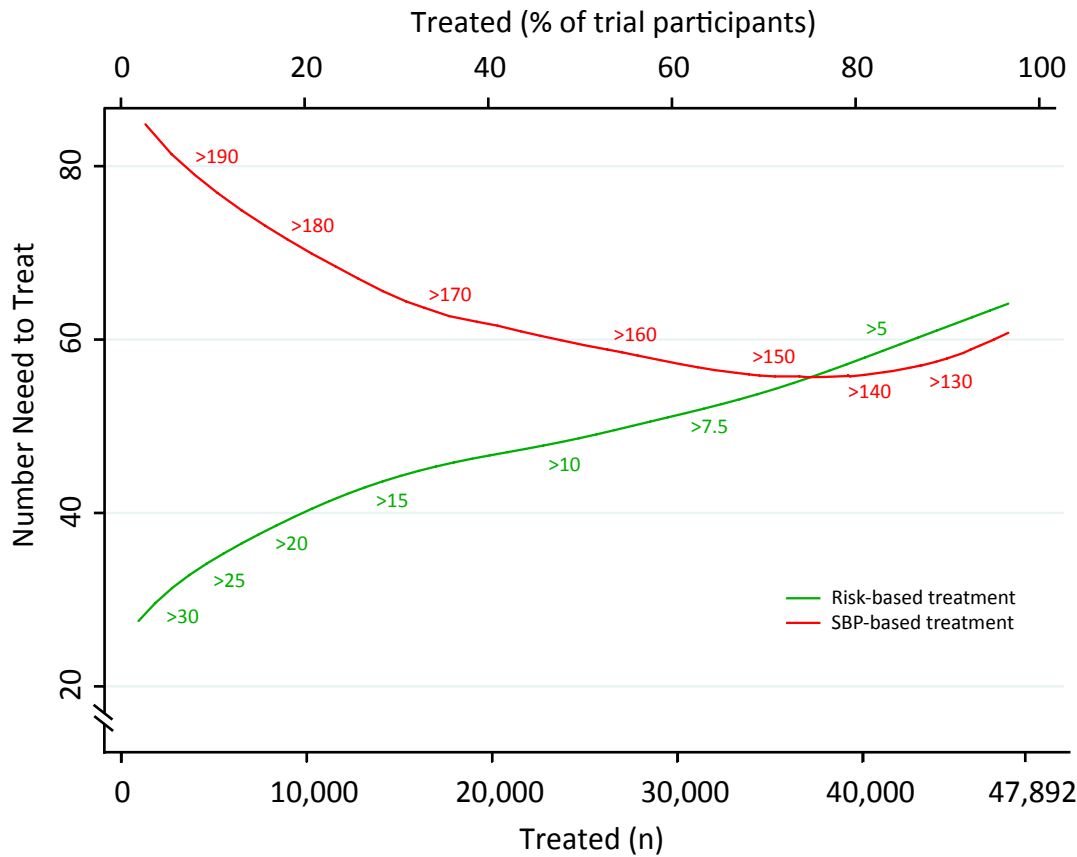

Supplement: S3 Fig — Numbers needed to treat for 5 y to avoid 1 cardiovascular event as a function of number of persons and proportion of sample treated using a CVD risk strategy (in green) and an SBP strategy (in red), standardized to a 5-mmHg SBP reduction. Numbers associated with each curve represent the specific CVD risk level (percentage 5-y CVD risk) or SBP (mmHg) at the treatment threshold. CVD, cardiovascular disease; SBP, systolic blood pressure. (PDF) [file pmed.1002538.s005.pdf]

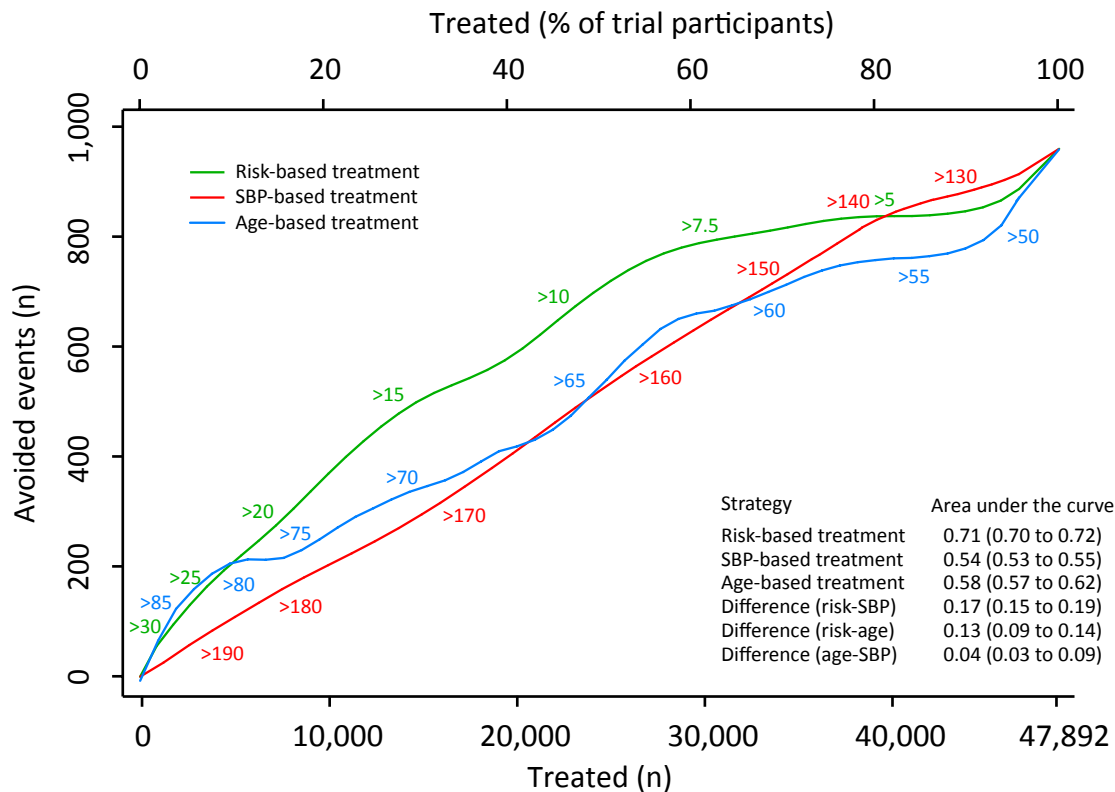

Supplement: S4 Fig — Expected cardiovascular events avoided over 5 y as a function of number of persons and proportion of sample treated using a CVD risk strategy (in green), an SBP strategy (in red), and an age-based strategy (in blue). Numbers associated with each curve represent the specific CVD risk (percentage 5-y CVD risk), SBP (mmHg), or age (y) level at that treatment threshold. CVD, cardiovascular disease; SBP, systolic blood pressure. (PDF) [file pmed.1002538.s006.pdf]

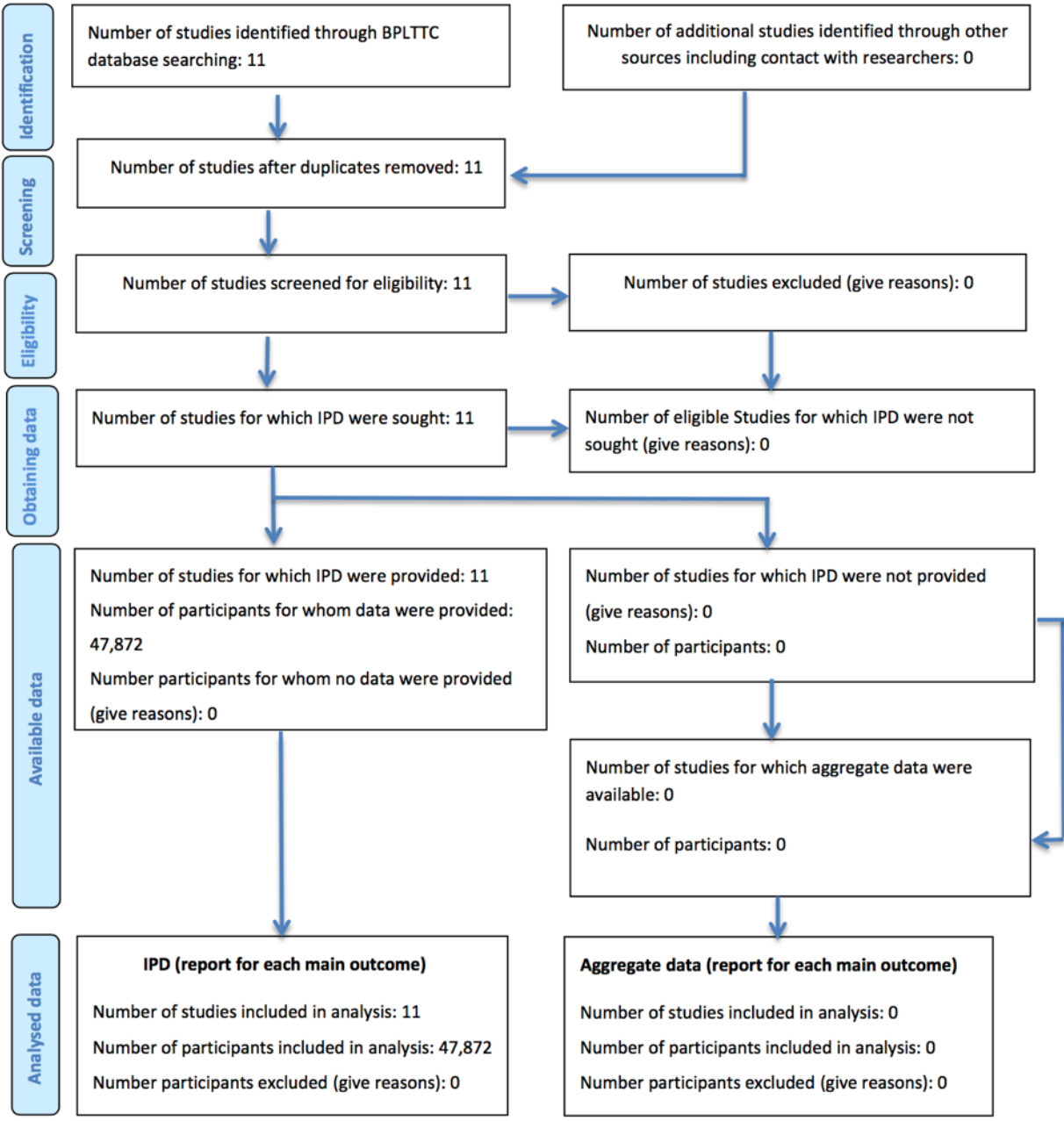

Supplement: S5 Fig — (PDF) [file pmed.1002538.s007.pdf]
